# Supplementary material for: Anti-prothrombin autoantibodies enriched after infection with SARS-CoV-2 and influenced by strength of antibody response against SARS-CoV-2 proteins
Source: PLoS Pathog. 2021 Dec 3;17(12):e1010118. doi: 10.1371/journal.ppat.1010118 (PMC8673606; doi:10.1371/journal.ppat.1010118)
Supplement: S1 Table — The best model is shown in bold letters (AIC, Akaike information criterion; β2, β2-glycoprotein I; DPO, day post onset; PC, principal component). (DOCX) [file ppat.1010118.s005.docx]

**S1 Table**

| **Equation** | **AIC** | **Log-likelihood** | $R_{adj}^{2}$ | **Likelihood ratio (p-value)** |
| --- | --- | --- | --- | --- |
| β2 IgM ~ 1 | 855.08 | -426.54 | 0 | - |
| **β2 IgM ~ 1 + PC1-SARS-CoV-2-IgG** | 845.76 | -420.88 | 0.110 | 11.32 (<0.001) |
| β2 IgM ~ 1 + PC1-SARS-CoV-2-IgG + (1 \| Sex) | 849.76 | -420.88 | 0.110 | 0 (ns) |
| β2 IgM ~ 1 + PC1-SARS-CoV-2-IgG + (PC1-SARS-CoV-2-IgG - 1 \| Sex) | 849.76 | -420.88 | 0.110 | 0 (ns) |
| β2 IgM ~ 1 + severity + PC1-SARS-CoV-2-IgG | 849.69 | -420.84 | 0.101 | 0.78 (ns) |
| β2 IgM ~ 1 + Age + PC1-SARS-CoV-2-IgG | 849.16 | -420.58 | 0.106 | 0.6 (ns) |
| β2 IgM ~ 1 + DPO + PC1-SARS-CoV-2-IgG | 681.23 | -336.61 | 0.003 | 168.53 (<0.001) |
| PT IgM ~ 1 | 700.69 | -349.35 | 0 | - |
| PT IgM ~ 1 + PC1-SARS-CoV-2-IgG | 678.15 | -337.08 | 0.279 | 24.5 (<0.001) |
| PT IgM ~ 1 + PC1-SARS-CoV-2-IgG + (1 \| Sex) | 677.29 | -334.64 | 0.353 | 4.87 (0.027) |
| PT IgM ~ 1 + PC1-SARS-CoV-2-IgG + (1 \| Sex) + (PC1-SARS-CoV-2-IgG -1\| Sex) | 675.09 | 332.55 | 0.417 | 4.19 (0.041) |
| PT IgM ~ 1 + PC1-SARS-CoV-2-IgG + severity + (1 \| Sex) + (PC1-SARS-CoV-2-IgG -1\| Sex) | 674.52 | 331.26 | 0.433 | 2.57 (ns) |
| PT IgM ~ 1 + PC1-SARS-CoV-2-IgG + severity + Age + (1 \| Sex) + (PC1-SARS-CoV-2-IgG -1\| Sex) | 673.41 | 329.71 | 0.450 | 5.68 (ns) |
| **PT IgM ~ 1 + PC1-SARS-CoV-2-IgG + DPO + (1 \| Sex) + (PC1-SARS-CoV-2-IgG -1\| Sex)** | 559.16 | 273.58 | 0.439 | 117.94 (<0.001) |
