# Supplementary material for: Anti-prothrombin autoantibodies enriched after infection with SARS-CoV-2 and influenced by strength of antibody response against SARS-CoV-2 proteins
Source: PLoS Pathog. 2021 Dec 3;17(12):e1010118. doi: 10.1371/journal.ppat.1010118 (PMC8673606; doi:10.1371/journal.ppat.1010118)
Supplement: S2 Table — The best model is shown in bold letters (AIC, Akaike information criterion; DPO, day post onset; PC, principal component; PT, prothrombin). (DOCX) [file ppat.1010118.s006.docx]

**S2 Table**

| **Equation** | **AIC** | **Log-likelihood** | $R_{adj}^{2}$ | **Likelihood ratio (p-value)** |
| --- | --- | --- | --- | --- |
| β2 IgM ~ 1 | 658.50 | -328.25 | 0 | - |
| **β2 IgM ~ 1 + PC1-SARS-CoV-2-IgG** | 650.87 | -323.43 | 0.134 | 9.63 (0.002) |
| β2 IgM ~ 1 + PC1-SARS-CoV-2-IgG + (1 \| Sex) | 654.87 | -323.43 | 0.121 | 0 (ns) |
| β2 IgM ~ 1 + PC1-SARS-CoV-2-IgG + (PC1-SARS-CoV-2-IgG - 1 \| Sex) | 656.87 | -323.43 | 0.121 | 0 (ns) |
| β2 IgM ~ 1 + severity + PC1-SARS-CoV-2-IgG | 658.84 | -323.42 | 0.107 | 0.03 (ns) |
| β2 IgM ~ 1 + Age + PC1-SARS-CoV-2-IgG | 659.84 | -322.92 | 0.106 | 1.02 (ns) |
| β2 IgM ~ 1 + DPO + PC1-SARS-CoV-2-IgG | 483.6 | -235.80 | 0.1 | 175.27 (<0.001) |
| PT IgM ~ 1 | 517.13 | -257.56 | 0 | - |
| PT IgM ~ 1 + PC1-SARS-CoV-2-IgG | 498.56 | -247.28 | 0.313 | 20.57 (<0.001) |
| PT IgM ~ 1 + PC1-SARS-CoV-2-IgG + (1 \| Sex) | 499.75 | -245.88 | 0.38 | 2.80 (ns) |
| **PT IgM ~ 1 + PC1-SARS-CoV-2-IgG + (1 \| Sex) + (PC1-SARS-CoV-2-IgG -1\| Sex)** | 496.9 | -243.45 | 0.48 | 7.66 (0.006) |
| PT IgM ~ 1 + PC1-SARS-CoV-2-IgG + severity + (1 \| Sex) + (PC1-SARS-CoV-2-IgG -1\| Sex) | 498.86 | -243.43 | 0.47 | 0.04 (ns) |
| PT IgM ~ 1 + PC1-SARS-CoV-2-IgG + severity + Age + (1 \| Sex) + (PC1-SARS-CoV-2-IgG -1\| Sex) | 497.77 | -241.88 | 0.49 | 3.13 (ns) |
| PT IgM ~ 1 + PC1-SARS-CoV-2-IgG + DPO + (1 \| Sex) + (PC1-SARS-CoV-2-IgG -1\| Sex) | 384.96 | -186.48 | 0.40 | 113.94 (<0.001) |
