# Supplementary material for: Anti-prothrombin autoantibodies enriched after infection with SARS-CoV-2 and influenced by strength of antibody response against SARS-CoV-2 proteins
Source: PLoS Pathog. 2021 Dec 3;17(12):e1010118. doi: 10.1371/journal.ppat.1010118 (PMC8673606; doi:10.1371/journal.ppat.1010118)
Supplement: S3 Table — The best model is shown in bold letters (AIC, Akaike information criterion; DPO, day post onset; PC, principal component; PT, prothrombin). (DOCX) [file ppat.1010118.s007.docx]

**S3 Table**

| **Equation** | **AIC** | **Log-likelihood** | $R_{adj}^{2}$ | **Likelihood ratio (p-value)** |
| --- | --- | --- | --- | --- |
| β2 IgM ~ 1 | 677.5 | -337.75 | 0 | - |
| β2 IgM ~ 1 + PC1-SARS-CoV-2-IgG | 677.39 | -336.70 | 0.015 | 2.10 (ns) |
| β2 IgM ~ 1 + PC1-SARS-CoV-2-IgG + (1 \| Sex) | 681.50 | -337.75 | 0.000 | 0 (ns) |
| β2 IgM ~ 1 + PC1-SARS-CoV-2-IgG + (PC1-SARS-CoV-2-IgG \| Sex) | 681.40 | -336.7 | 0.016 | 2.10 (ns) |
| β2 IgM ~ 1 + severity + PC1-SARS-CoV-2-IgG | 680.46 | -336.23 | 0.014 | 3.04 (ns) |
| β2 IgM ~ 1 + Age + PC1-SARS-CoV-2-IgG | 681.16 | -336.58 | 0.004 | 2.34 (ns) |
| β2 IgM ~ 1 + DPO + PC1-SARS-CoV-2-IgG | 681.23 | -336.61 | 0.003 | 2.27 (ns) |
| PT IgM ~ 1 | 578.95 | -288.48 | 0 | - |
| PT IgM ~ 1 + PC1-SARS-CoV-2-IgG | 565.83 | -280.91 | 0.213 | 15.13 (<0.001) |
| PT IgM ~ 1 + PC1-SARS-CoV-2-IgG + (1 \| Sex) | 563.67 | -277.83 | 0.328 | 6.16 (0.013) |
| PT IgM ~ 1 + PC1-SARS-CoV-2-IgG + (1 \| Sex) + (PC1-SARS-CoV-2-IgG -1\| Sex) | 561.19 | -275.6 | 0.402 | 4.48 (0.034) |
| **PT IgM ~ 1 + PC1-SARS-CoV-2-IgG + severity + (1 \| Sex) + (PC1-SARS-CoV-2-IgG -1\| Sex)** | 558.67 | -273.34 | 0.446 | 4.52 (0.033) |
| PT IgM ~ 1 + PC1-SARS-CoV-2-IgG + severity + Age + (1 \| Sex) + (PC1-SARS-CoV-2-IgG -1\| Sex) | 557.93 | -271.97 | 0.464 | 2.74 (ns) |
| PT IgM ~ 1 + PC1-SARS-CoV-2-IgG + DPO + (1 \| Sex) + (PC1-SARS-CoV-2-IgG -1\| Sex) | 560.14 | -273.07 | 0.442 | 0.53 (ns) |
