# Supplementary material for: Anti-prothrombin autoantibodies enriched after infection with SARS-CoV-2 and influenced by strength of antibody response against SARS-CoV-2 proteins
Source: PLoS Pathog. 2021 Dec 3;17(12):e1010118. doi: 10.1371/journal.ppat.1010118 (PMC8673606; doi:10.1371/journal.ppat.1010118)
Supplement: Attachment — Submitted filename: 118156_1_rebuttal_2161537_qyhvk8.docx [file ppat.1010118.s009.docx]

**Reviewer #2 (Evidence, reproducibility and clarity (Required)):**

This paper attempts to address a current, clinically relevant question utilizing novel statistical modeling. The authors comprehensively assessed the presence of criteria and non-criteria aPL in a heterogeneous cohort of 75 COVID patients and 20 non-infected controls. They found 66% of COVID patients had positive aPL and demonstrated a correlation between aPL and anti-SARS-CoV-2. However, I have several major concerns:

1. The cohort is extremely heterogeneous. COVID-19 samples that were used included hospitalized patients and those who had COVID more than 2 months ago and were convalesced (29% of samples). Severity of disease does influence autoreactivity and the presence of autoantibodies. The prevalence of autoantibodies among patients who are acutely ill will be much different than those who are convalesced. I think it would be prudent to assess the presence and correlation of aPL among those two groups separately.

We thank you for pointing out the complexity of our study population, consisting of multiple cohorts from different centres. Exactly the above-mentioned heterogeneity of our cohorts and their variables is the reason why we employed linear mixed-effect models. Linear mixed-effect smodels, accounting for both fixed as well as random effects, are suitable to address potentially confounding factors. Along these lines, disease severity (different in the convalescent and the acutely ill individuals) as well as the relation of the time of sampling to time of disease occurrence (days post onset of disease manifestation) were included as fixed effects in our mixed model. Thus, our model accounts for potential differences between the acute phase of infection and convalescent phase and would capture them if relevant.

In order to increase the rigour, we have performed an additional analysis where we excluded the convalescent individuals from the model (see **Fig. 3C**). The results obtained are in line with results already shown (**Fig. 3B, 3D**).

In general, we have pursued a largely data-driven exploratory, and not a hypothesis-driven, approach. Clearly, we could have decided to set a stringent focus on a cohort without complexity. Yet, our approach encourages heterogeneity, which we address using an adequate model. Since, perhaps, the model choice, the model itself, and the data-driven approach were not explained extensively enough, we have added a more detailed account in the manuscript, lines 317-334 and lines 394-403.

2. Sampling of the patients is concerning, 35% are plasma and 65% are serum. It is undesirable to put data from plasma and serum together to perform analysis.

We thank the reviewer for raising this important concern. We have aimed to be as rigorous and transparent as possible in the description of the cohorts (see **Tables 1** and **2**) for serum/plasma). While we agree that, in general, it would be best if either only plasma (i.e., only heparin plasma or only EDTA plasma) or only serum was used, the authors wish to clarify that for both SARS-CoV-2 IgG profiling as well as for LIA, plasma or serum can be used interchangeably. We can formally show this. We have conducted a SARS-CoV-2 IgG profiling experiment on patient-matched samples (plasma and serum). Data is unambiguous about that there is no effect of plasma or serum on the assay outcome (**Fig. S3A** and **S3B**), with a Pearson correlation coefficient of 0.9942 (95% confidence interval: 0.9865-0.9975) and R2 of 0.9885. Bland-Altman analysis does not indicate any significant bias (**Fig. S3C**).

For the detection of APS antibodies with ELISA, literature is suggestive of no relevant interference by the usage of plasma or serum on the measured value (Pham *et al.*, 2019). To formally reassess this, we measured aPL autoantibodies with LIA in one matched plasma and serum sample of an individual with high-titre aPL antibodies and of one high-titre individual whose plasma was spiked into non-reactive plasma and serum (**Fig. S2A** and **Fig. S2B**). We found the same pattern of IgM and IgG aPL-positivity in both matched serum and plasma samples as well as in spiked serum and plasma samples, with a Pearson correlation coefficient of 0.9974 (95% confidence intervals: 09611-1.034) and R^2^ of 0.9813 (**Fig. S2A**). Bland-Altman analysis did not indicate a significant bias (**Fig. S2B**).

We therefore conclude that in our study, using both plasma as well as serum has no effect on the validity of our results.

3. LIA based assays were used to assess the presence of aPL and results were reported in OD rather than standardized units. While the same group demonstrated a positive correlation in the past between LIA OD and internationally accepted ELISA-based aPL assays, the validity and clinical utility of these LIA assays still require further evaluation. Furthermore, OD>50 was used as a positive cut-off. How this cut-off was determined and how it relates to internationally accepted positive aPL cut-offs (99th percentile or greater than 40) remains unclear.

We thank the reviewer for mentioning concerns on LIA. The validity of this technology has been confirmed in multiple peer-reviewed publications (Roggenbuck et al. Arthr Res Ther 2016;18:11, Nalli et al. Autoimmunity Highlights 2018;9,6). In terms of cut-off detection, processed strips were analysed densitometrically employing a scanner with the evaluation software Dr. DotLine Analyzer (GA Generic Assays GmbH). The cut-off of 50 OD units was determined by calculating the 99th percentile of 150 apparently healthy individuals as recommended by the international classification criteria for aPL testing and Clinical and Laboratory Standards Institute (CLSI) guideline C28-A3 (Roggenbuck et al. Arthr Res Ther 2016;18:11, Nalli et al. Autoimmunity Highlights 2018;9,6). A corresponding sentence has been added to the METHODS AND MATERIALS section.

For our study, we aimed to perform the maximum number of tests possible with limited sample volume and have therefore chosen LIA. We are aware of the discussion on internationally accepted cut-offs for clinical APS diagnostics. However, we would like to point out that our manuscript is not a case report on patients diagnosed with APS, nor do we aim to modify diagnostic standards set in the international consensus statement for the classification criteria for definite APS (established in 2006).

Moreover, the OD ≥ 50 was used as a cut-off in one analysis (with Fisher’s exact test for statistics) in our manuscript and was re-assessed using Mann-Whitney/Wilcoxon rank sum test on a continuous scale (**Fig. 1C** and **1D**). All subsequent analyses were not contingent on an OD cut-off. We believe that this is clearly stated in the manuscript.

4. While the authors attempted to evaluate the presence of both IgG and IgM aPL in COVID patients, only 65% of samples were tested for both IgG and IgM aPL.

We agree that testing the entire collective for IgG and IgM isotypes would have been best. In fact, we would have been interested in also including the IgA isotype. Inconveniently, sample volume is sometimes limiting.

We have been clear about the omission of IgG aPL measurements in the samples from Zurich (see lines 214-215). We consider this a limitation, however, our data indicated that IgM aPLs are more immediately relevant in the context of SARS-CoV-2. While this has been surprising to us, we would like to highlight that this is a manifestation of the quality of a data-driven approach where data, much more than belief, build the foundation for conclusions. Along these lines, we could have easily omitted all data on IgG aPLs without compromising the message contained in our manuscript. However, we stand behind our decision to show all data even if, in the case of IgG aPL, (1) they are mostly negative and (2) they are incomplete.

5. 26 patients had anti-SARS-CoV-2 data already available. Whether those were tested on the same samples and at the same time points as aPL ais not clear.

We apologise for not having been clear about this in the text. The 26 samples from Zurich had been included in another study where their respective anti-SARS-CoV-2 Spike ECD, RBD, and NC p(EC50) values were used (Emmenegger *et al.*, 2020). Thus, the p(EC50) values have been re-used in the current manuscript. The aPL autoantibodies were measured on exactly the same samples. We have tried to improve the explanation of this in the text, see lines 300-301.

6. The novel statistical modelling design is interested. However, as there are concerns about the data put into the modelling, the validity of the conclusions is debatable.

We thank the reviewer for being interested in the statistical model we used. Linear regression analysis belongs to the standard equipment when performing epidemiological analyses (see e.g., Szklo, Nieto, Epidemiology: Beyond the Basics). Here, we have employed a linear mixed-effects model to infer changes in the predictive power of fixed and random variables (e.g. SARS-CoV-2 IgG levels, disease severity, age, sex, days post onset of disease manifestation), to determine which of these variables reliably predict an outcome (e.g. PT aPL levels), and in what combination.

We recognised that the manuscript would benefit from a more thorough explanation of the model and how it helps to evaluate the validity of the data. We have therefore added lines 317-334 in the manuscript.

All authors are appreciative of the reviewer’s critique. In the light of the answers we provided, we are convinced about our conclusions, based on the data and our dataset. We hope that, with our responses, we have adequately addressed the concerns raised by the reviewer.

**Reviewer #2 (Significance (Required)):**

See above.


**Reviewer #3 (Evidence, reproducibility and clarity (Required)):**

It is being recognized that SARS-CoV-2 infection leads to acquired thrombophilia with increased arteriovenous thrombosis and endothelial injury and organ damage. This has multiple mechanisms including, the hypercoagulable state with platelet activation, endothelial dysfunction, increased circulating leukocytes, cytokines and fibrinogen, but also the acquired thrombophilia could be due to acquired APS in these patients. In this study, Emmenegger et al. evaluated aPL antibody responses in SARS-CoV2 infected individuals in connection with antibodies against the SARS-CoV2 components and found that antibody strength response against SARS-CoV-2 proteins is associated with PT IgM aPL antibody.

**Reviewer #3 (Significance (Required)):**

This is overall an interesting and thought-provoking study, as it may explain the development of thrombophilia after SARS-CoV-2 vaccination. While the study provides a possible association of the development of antibodies against SARS-CoV-2 infection and aPL, it does not go to molecular details about the homology between anti- SARS-CoV-2 antibodies and aPL. Therefore, the study remains an association study.

First of all, we would like to thank the reviewer for the careful evaluation of our work. We are in full consciousness of the descriptive nature of our work. Thanks to the suggestion of the reviewer (see below), we have aimed to go one step further into a more functional/ mechanistic description.

It is not surprising that they found a difference in IgM rather than IgG as IgM development is an early response.

The overall conclusion is supported by the rigorous statistical analyses, yet the study remains a correlative and association study.

Significance: Thrombophilia associated SARS-CoV2 may be due to immunity against SARS-CoV2 rather than that pure cytokine response.

Furthermore, they did not characterize the PT IgM aPL to find which part could be immunogenic or epitope similarity with anti- SARS-CoV-2 antibodies. Identification of these epitopes is crucial for further understanding of the antibody development and further intervention.

Existing literature does not connect with antibody responses against Sars-CoV2.

Could the authors provide some molecular epitope analysis of IgM aPl and ani Sars_ antibodies? Even computation analysis will improve the paper tremendously.

We thank the reviewer for coming up with this idea. Clearly, the presence of cross-reactive IgM antibodies to human prothrombin, triggered against the SARS-CoV-2 Spike protein, would be a direct and simple explanation for our observation. We have put efforts into analysing epitopes of SARS-CoV-2 Spike protein and prothrombin (see lines 374-390 in the manuscript and **Fig. 4)**. We conclude there is very limited similarity, and that the mechanism is most likely indirect.

There is no ethical concern.
