# Supplementary material for: Anti-prothrombin autoantibodies enriched after infection with SARS-CoV-2 and influenced by strength of antibody response against SARS-CoV-2 proteins
Source: PLoS Pathog. 2021 Dec 3;17(12):e1010118. doi: 10.1371/journal.ppat.1010118 (PMC8673606; doi:10.1371/journal.ppat.1010118)
Supplement: Attachment — Submitted filename: Reply_reviewersPlosPathogens_kf.docx [file ppat.1010118.s010.docx]

Dear editors, dear reviewers,

We thank you for your favourable and critical assessment of our manuscript. We have addressed all your considerations and incorporated changes in the manuscript.

The two main points of discussion have been (1) the validity of data incorporated in the statistical model and (2) the time-dependence of humoral immune responses.

In our study, we have chosen a multi-step data-driven approach (mentioned in lines 236-237 and 277-278 and 301-309 and 350-369 and 442-449 and 567-568). Essentially, we have collected data from multiple cohorts and, following a descriptive characterisation of the cohort (lines 198-216, **Table1** and **Table 2**, **Fig. S1**), first explored the data, without formulating a clear hypothesis (lines 235-255, **Fig. 1A/B**).

Until this stage of the analysis, we have aimed to be maximally inclusive. Prior knowledge is limited or even non-existent. It could be that upon infection with SARS-CoV-2, an aPL immune response against one or multiple aPL is elicited, or against none. The temporal rise of such a response could be even more immediate than against viral proteins, or the opposite may be the case. Further, the kinetics of the IgM response, if detectable, and its seroconversion into IgG, is unknown. Thus, there was no reason to exclude data from analysis at this point of time. We may subsequently find that there is no trace of either IgM or IgG aPL, against any of the aPLs tested. Or we would find that all aPL are elevated upon infection, independent of time, but only regarding IgG and not IgM. This was the initial hypothesis-generating step of our data-driven approach. Accordingly, this will required an elaborate follow-up.

Therefore, as a starting point, we asked whether there was any difference in aPL IgM or IgG levels in infected versus non-infected individuals (lines 256-276, **Fig. 1C/D**), while appreciating the fact that our data were heterogeneous (lines 236-237). Using two conventional statistical models (using data binarization and non-binarized data), we found that β2-glycoprotein and prothrombin IgM aPL were enriched in the infected versus non-infected individuals (**Table 3** and **Fig. 1C/D**).

This finding was subsequently challenged by investigating potential bias, as there was a difference in the sex and age distribution between the infected cohorts and the non-infected controls (lines 277-300, **Fig. S1**). We found that neither age nor sex were confounders. Compared to the previous version of our manuscript – and thanks to the constructive suggestions by the reviewers – we more carefully distinguished between the convalescent individuals and those with acute infection (see lines 257-259, **Fig. 1** and **Fig. 2**), already at this stage. As we indeed found evidence, although limited, for IgG seroconversion at late DPO (lines 241-242, **Fig. 1B/D**), we now discuss this in the manuscript (491-502). Nervetheless, in our study, we have decided to focus on β2 as well as PT IgM for further analyses.

Next, we wanted to investigate, in depth, the relationship between the aPL IgM response against β2 and PT (lines 370-372) and all available parameters, additionally considering the IgG response against SARS-CoV-2 proteins (lines 310-346, **Fig. 2**, **Fig. S2**). We chose a linear mixed-effects model (LMM), explained in quite some detail (lines 350-369 and 441-449) and followed a manual step-up approach. In addition, we illustrated relationships by means of three-dimensional representations (two coordinates, one colour-coded, see **Fig. 3A**,), however, the statistical model accounted for all dimensions at the same time.

The conclusions of the model, i.e., the dependency of β2 IgM but mostly PT IgM aPL levels on the strength of the anti-SARS-CoV-2 antibody response and the additional dependency of PT aPL levels on sex (lines 399-400, **Fig. 3B**, **Table S1**) was confirmed upon exclusion of the convalescent individuals and the healthy donors (lines 405-420, **Fig. 3C/D**, **Table S2/3**).

We have included additional sections discussing the limitations of our study, including how to examine the IgG response in our cohort (see lines 491-502). Our investigation is not and cannot be entirely holistic. We started with a multitude of parameters (allowing us and the readers of the manuscript to gain a broad overview), which we described extensively and transparently. We then made few observations and followed up on several of them, while others required additional studies. In this light, we consider our study to be a starting point for further investigations.

Reviewer Comments (if any, and for reference):

Reviewer's Responses to Questions

**Part I - Summary**
Please use this section to discuss strengths/weaknesses of study, novelty/significance, general execution and scholarship.

Reviewer #1: I'd like to thank the authors for addressing my concerns with added data and text. I think the manuscript has significantly improved. I do not have any further comments.

We kindly thank Reviewer 1 for the critical input during the first round of the reviewing process.

Reviewer #2: Summary: This study investigated the presence of antiphospholipid antibodies (aPL) in different stages of COVID-19 in different patients at different stages of disease or recovery. The aetiology of hypercoagulability in COVID-19 is a pressing question given the severe morbidity and mortality that arises and the need to find appropriate treatments. Research in the field is important.

The study has already been reviewed once, and the authors have responded, where possible, to some of the reviewers’ comments. This is evidenced by demonstration of the interchangeable use of serum or plasma in serology assays, and the sequence analysis of SARS-CoV-2 spike and human prothrombin. However, the original limitations in the study design and the interpretation of data that are associative rather than tractable remain weaknesses in the manuscript.

We thank Reviewer 2 for bringing these important topics to our attention. We are conscious about the limitations in our study, including study design, and the associative nature of our findings. As shown above and further below, we have more clearly shaped out the rationale behind the study design and explicitly discussed the limitations in our approach.

**Part II – Major Issues: Key Experiments Required for Acceptance**
Please use this section to detail the key new experiments or modifications of existing experiments that should be absolutely required to validate study conclusions.

Generally, there should be no more than 3 such required experiments or major modifications for a "Major Revision" recommendation. If more than 3 experiments are necessary to validate the study conclusions, then you are encouraged to recommend "Reject".

Reviewer #1: (No Response)

Reviewer #2: As pointed out by reviewer number 2, the cohort is highly heterogenous. Combining the analysis of sera/plasma from individuals who have survived COVID-19 and who are in the convalescent phase (and may therefore bear features of survival) with those suffering from acute illness is illogical. Serological diagnosis is time dependent and this forms the basis of acute and convalescent testing in infectious disease. Furthermore, induction of IgM and IgG is time sensitive. The time dependency of humoral responses is observable in Figure 3A where OD of PT IgM, PC1 SARS-CoV-2 IgG and B2Igm are generally lower in those further from disease onset. The lack of a relationship between aPL IgG and SARS-CoV-2 -specific antibody combined suggests a transient acute response. These features of serological evolution have not been appropriately considered in the study design.

It is a fact that for the first part of the analysis, we have included convalescent as well as acutely infected individuals and grouped them into ‘SARS-CoV-2 infected’. They have then been compared to the non-infected control group. We could have simply omitted the 22 individuals pertaining to the convalescent cohort altogether and conducted the entire study without them. In hindsight, we would not have lost much information. However, prior knowledge did not allow us, at that stage, to appreciate which feature is truly important and which is not, as explained above. The distinction had been made already in the previous version using the LMM (see **Fig. 3**). However, we have included two major changes as we agree with the Reviewer that comparisons must be done with utmost care:

1. We have colour-coded the convalescent individuals and the acutely infected in **Fig. 1B/C/D** as well as in the heatmaps (**Figs. 1A**, **2A**), to better appreciate whether there is any difference between the two. We have additionally provided supplementary figures (**Figs. XXX**) that help clarify why, for further analyses, we have chosen an LMM.

2. We improved the discussion in that we clearly mentioned the limitations in our study, also regarding aPL IgG (lines 491-502). Importantly, our observations have helped narrow down from a dozen parameters to few parameters. Follow-up studies can focus on findings that we report, without requiring the same level of width, going into more details.

Whilst a statistical model has been used to try to unpick heterogeneity, concise clear case definition, alongside timing of diagnosis and PCR testing prior to data analysis would be preferable. Table 3 has not been divided into the two groups amongst data from those defined as SAR-CoV-2 infected. Reviewer #2 point 6 that there are concerns about the data put into the modelling, which brings into question the validity of the conclusions remains unresolved.

We have provided a detailed answer to this comment already above in our current replies, in the manuscript, as well as in the last round of replies sent to reviewers. Based on previous as well as on current improvements, explained above, the validity of both data sets as well as conclusions drawn thereof can be guaranteed. In fact, there are few manuscripts in which statistical findings are pursued as much as in ours, using a multitude of statistical models that are challenged within the same study. We could have streamlined the study by omitting data (e.g., the convalescent individuals), thereby decreasing heterogeneity. However, our study embraces heterogeneity and uses appropriate models to account for it. Based on insights gained by us and others, follow-up investigations can be more focused and incorporate only concise clear case definitions, including rigorous homogenous sampling after PCR detection or after first symptoms.

Given the possibility that aPL are associated with the hypercoagulable state, case definition would be strengthened by an understanding of those where there was demonstrable clinical evidence of this e.g. pulmonary embolism.

We agree that an association between aPL and clinical evidence of thrombotic events, e.g., pulmonary embolism, would be valuable. Based on our findings, the design of such a study is facilitated: a cohort of patients having suffered from thrombotic events following infection with SARS-CoV-2 can be directly compared to a cohort of patients without evidence of thrombosis after infection with SARS-CoV-2, by looking at PT IgM and potentially PT IgG levels. However, we would like to mention that clinical signs of thromboembolism could be missed as reported in an autopsy study (Wichmann et al. 2020 Ann Intern Med. 2020 Aug 18;173(4):268-277. doi: 10.7326/M20-2003).

The data interpretation does not take account of literature demonstrating a link between induction of aPL and several different infections, including where a vasculitis or endothelial injury occurs. For example, infection with Treponema pallidum is associated with the induction of anticardiolipin antibodies which are used as a surrogate measure of infection, disease activity and re-infection. However, these patients do not typically enter a hypercoagulable state, possibly due to differences in binding specificity of antibodies in antiphospholipid syndrome compared with syphilis (Pierangeli S. et al 1994). It is possible for aPL antibodies to bind with no functional significance for clotting and causality cannot be assumed. The line immunoassay does not measure antibody function in this regard. Can further studies be done to assess this?

We thank the Reviewer for bringing up this important point. Our assay, the LIA, like other solid-phase assays used in the APS field, investigates binding. The functionality of antibodies where binding is observed is a topic that we do not address experimentally. The clinical diagnosis of APS (according to the international consensus statement on the classification of APS), including disease phenotype and presence of aPL (Myakis et al., 2006 J Thromb Haemost. 2006 Feb;4(2):295-306. doi: 10.1111/j.1538-7836.2006.01753.x.), is bound to the same limitation: the occurrence of aPL, shown in the binding assay, does not predict their function. However, the absence of binding in the binding assay precludes subsequent function, as the antibodies are not even there.

Figure 3A suggests a link with female sex and PT IgM. COVID-19 is more severe in men. This apparent paradox is not discussed. It is also surprising that no effect of age was observed given the striking linear association of age with mortality from COVID-19.

We have added a discussion on the influence of sex in the manuscript (lines 530-535). To better visualise the correlation with other features (for e.g., age), in addition to **Fig. 3A**, we have included **Fig. S4** and have commented on the observations (see lines 370-385).

Reviewer #3 comment that this study may explain the development of thrombophilia after SARS-CoV-2 vaccination is not valid. There is no vaccinated cohort in this dataset in which to study this phenomenon.

Given the literature indicating aPL in multiple unrelated infections, it is unsurprising that there is no homology between anti-SARS-CoV-2 antibodies and aPL. Although molecular mimicry is a possibility, it is also possible that release of host phospholipids through cellular damage could induce aPL. However, it is helpful that the authors have done some work to investigate this in Fig 4.

Thank you for the feedback. We have also considered it a less likely hypothesis that antibodies to SARS-CoV-2 cross react with the Spike protein, shown in **Fig. 4**. We have additionally performed the analysis for the nucleocapsid protein where we did not find homologous epitopes (data not shown). If the reviewers or editors consider this a worthwhile addition, although essentially negative, we would be open to adding the data to **Fig. 4**, requiring only minimal changes in the text.

Several other studies have investigated aPL in COVID-19 and these have been published with conflicting data and interpretation. The findings from this study should be placed in the context of what has already been published.

We have added recent literature and extended our discussion (see lines 466-478).

**Part III – Minor Issues: Editorial and Data Presentation Modifications**
Please use this section for editorial suggestions as well as relatively minor modifications of existing data that would enhance clarity.

Reviewer #1: (No Response)

Reviewer #2: Part III – Minor Issues: Editorial and Data Presentation Modifications
Please check the manuscript for wording and grammatical errors, particularly in the abstract.
