# Supplementary figures and images for: Correction: Anti-prothrombin autoantibodies enriched after infection with SARS-CoV-2 and influenced by strength of antibody response against SARS-CoV-2 proteins
Source: PLoS Pathog. 2022 Feb 2;18(2):e1010289. doi: 10.1371/journal.ppat.1010289 (PMC8809571; doi:10.1371/journal.ppat.1010289)

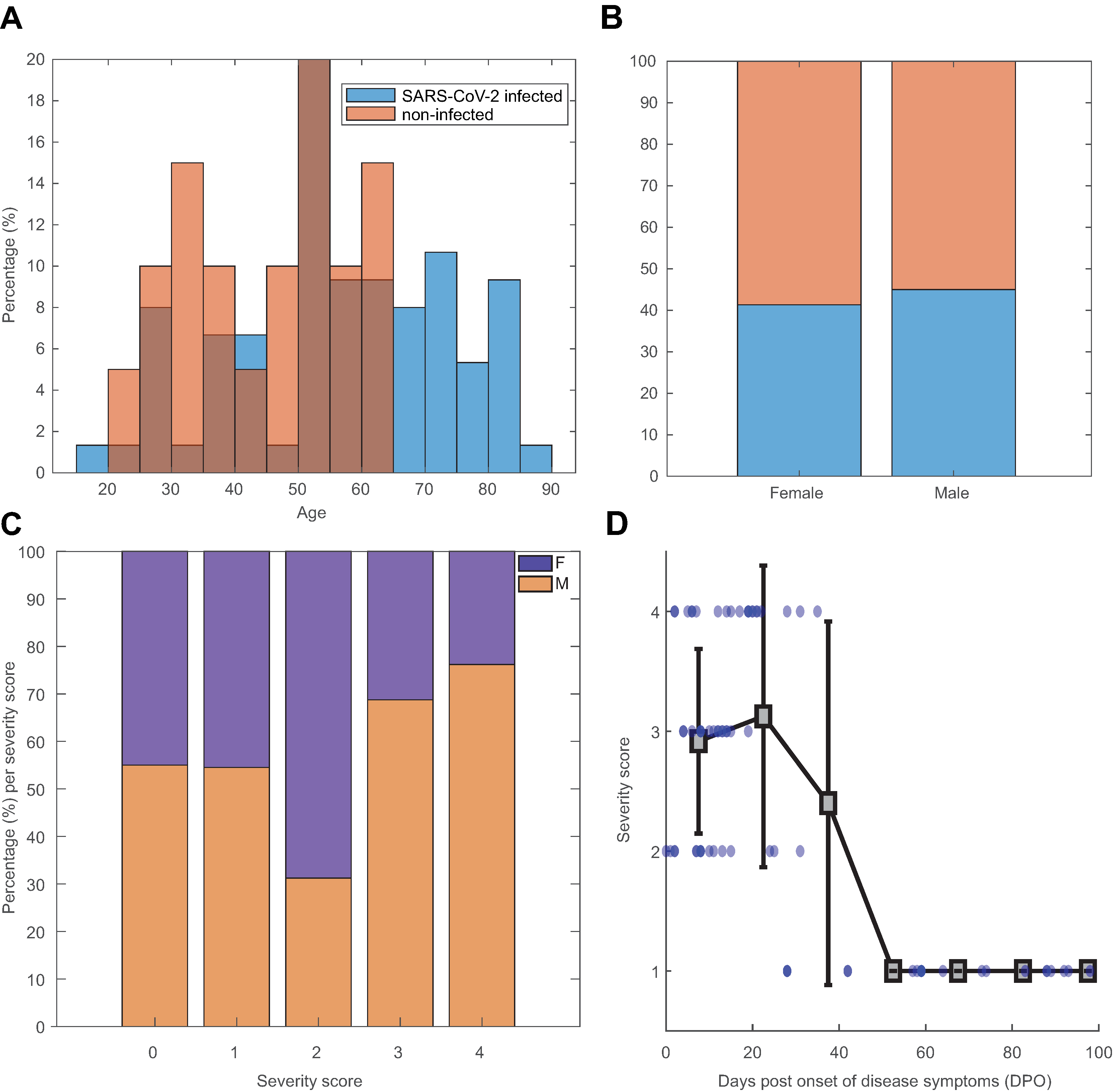

Supplement: S1 Fig — A. Age distribution of entire cohort for non-infected controls (orange) and SARS-CoV-2 infected individuals (blue). The distribution of the controls indicates a generally younger age versus the SARS-CoV-2 infected individuals. B. Sex distribution of entire cohort for non-infected controls (orange) and SARS-CoV-2 infected individuals (blue). The distribution in both cohorts was slightly skewed towards males versus females. C. Female (violet) and male (light orange) representation (in %) within each severity score. The distributions are slightly skewed towards more male than female individuals in all severity scores, except for score 2. D. Representation of DPO related to severity score. The convalescent individuals included in this study and sampled at later DPO all had a severity score of 1 while the acutely infected hospital patients had severity scores between 2–4. (TIF) [file ppat.1010289.s001.tif]

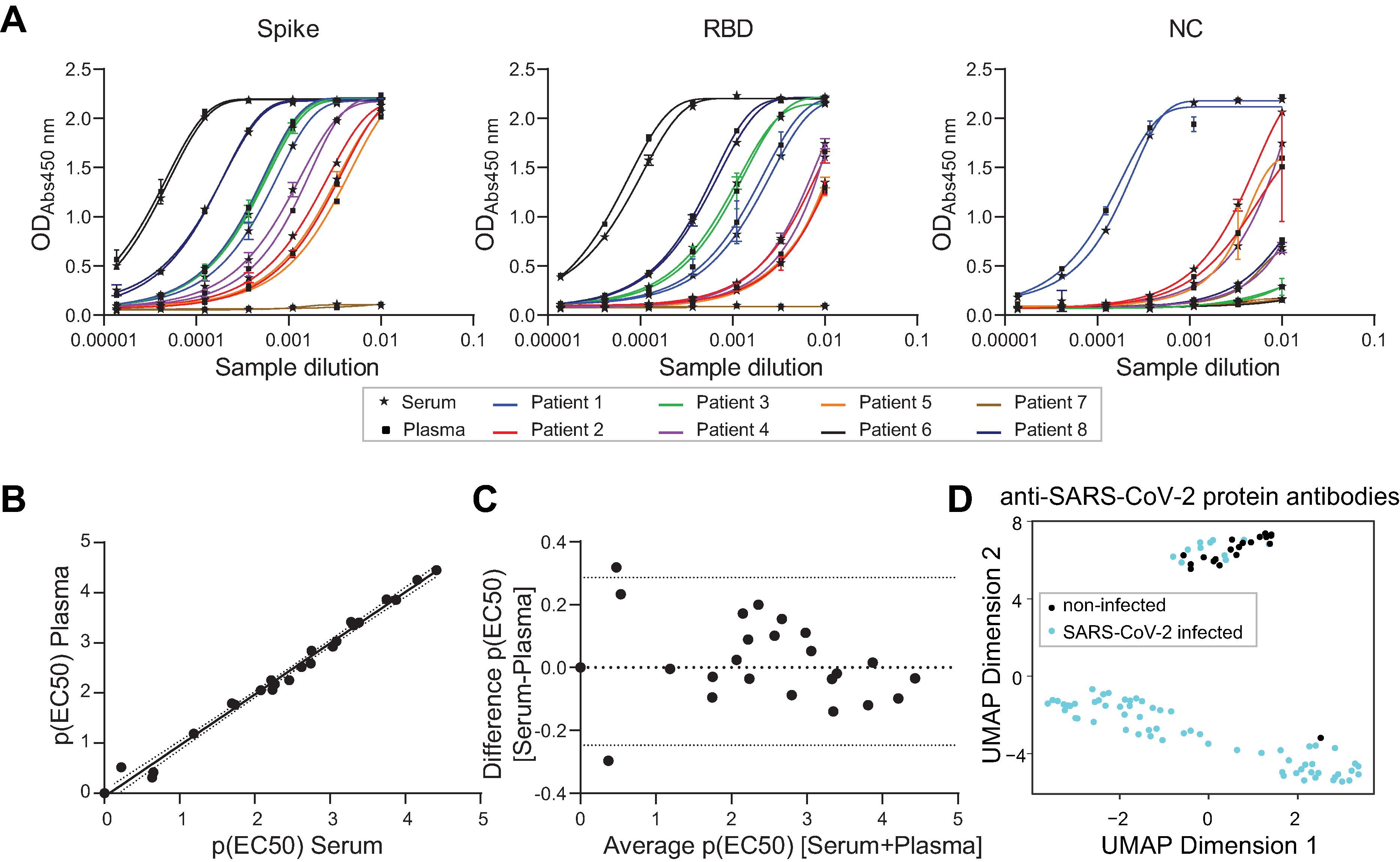

Supplement: S3 Fig — A. Binding curves of patient-matched serum and plasma samples. Three SARS-CoV-2 proteins were used to conduct the tripartite-autoimmune blood immunoassay technology: the SARS-CoV-2 spike ectodomain (S), its receptor binding domain (RBD), and the nucleocapsid protein (NC). All samples were tested as technical duplicates and are shown as mean with standard deviation. B. Based on the binding curves, the concentration at half-maximum binding of the sigmoidal curve, or the p(EC50) value, was calculated. p(EC50) values for S, RBD, and NC for all patients are compared in a scatter plot with serum-based p(EC50) values on the x-axis and plasma-based p(EC50) values on the y-axis. The Pearson correlation coefficient was 0.9942 (95% confidence interval: 0.9865–0.9975) and R2 was 0.9885. Dotted line represents the 95% confidence interval of the linear regression. C. Visualisation of the same data as shown in (B) using a Bland-Altman plot. Bias was calculated to be 0.01976 with a standard deviation of 0.1361. Dotted lines represent the confidence intervals (-0.2471 to 0.2866). D. UMAP representation of anti-SARS-CoV-2 protein antibodies displays clear clusters, with non-infected controls (black) and non-IgG-reactive SARS-CoV-2 infected individuals (turquoise) clustering separately from IgG-reactive SARS-CoV-2 infected individuals. (TIF) [file ppat.1010289.s002.tif]

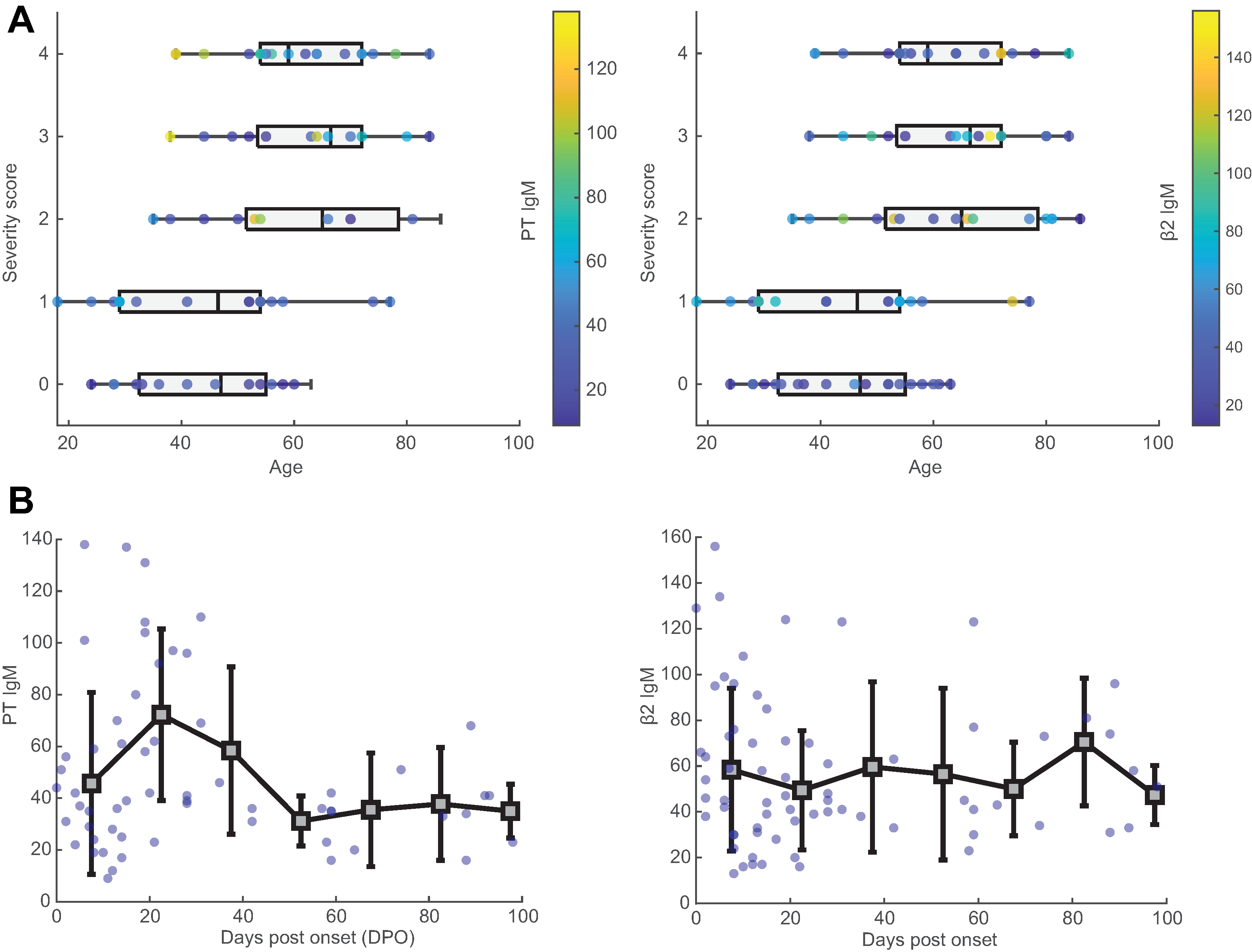

Supplement: S4 Fig — A. Severity score as a function of age, with colour-coded PT (left) and β2 (right) IgM levels. When ignoring the interdependency of features, data suggests that PT and β2 IgM levels are modulated by severity but not by age. B. PT and β2 IgM levels as a function of DPO. PT IgM aPL levels show a non-homogenous distribution along DPO, with a rise between DPO 10–30 and a subsequent drop. β2 IgM levels, on the other hand, seem stable over time. To summarize the data, aPL levels were binned in 15 day windows. The LMM used in this study takes care of the interdependency of parameters and accounts for redundancy, in the present case e.g., between severity score and DPO and their effect on PT or β2 IgM titres. Values are shown as single dots, with boxplot (median and interquartile range) in (A) and as mean ± standard deviation in (B). (TIF) [file ppat.1010289.s003.tif]
